# Evolutionary Analysis of Unicellular Species in Chlamydomonadales Through Chloroplast Genome Comparison With the Colonial Volvocine Algae

**DOI:** 10.3389/fmicb.2019.01351

**Published:** 2019-06-18

**Authors:** Yuxin Hu, Weiyue Xing, Huiyin Song, Huan Zhu, Guoxiang Liu, Zhengyu Hu

**Affiliations:** ^1^Key Laboratory of Algal Biology, Institute of Hydrobiology, Chinese Academy of Sciences, Wuhan, China; ^2^University of Chinese Academy of Sciences, Beijing, China; ^3^Key Laboratory of Marine Ecology and Environmental Sciences, Institute of Oceanology, Chinese Academy of Sciences, Qingdao, China; ^4^State Key Laboratory of Freshwater Ecology and Biotechnology, Institute of Hydrobiology, Chinese Academy of Sciences, Wuhan, China

**Keywords:** higher substitution rates, Chlamydomonadales, chloroplast genome, colonial volvocine algae, unicellular species, positive selection

## Abstract

This study is the first determination of six chloroplast genomes of colonial volvocine algae, *Colemanosphaera charkowiensis*, *Volvulina compacta*, *Pandorina colemaniae*, *Pandorina morum*, *Colemanosphaera angeleri*, and *Yamagishiella unicocca*. Based on 55 chloroplast protein-coding genes, we compared the nonsynonymous (dN) and synonymous (dS) substitution rates between colonial volvocine algae and the other unicellular Chlamydomonadales species. When refer to the dN, we found 27 genes were significantly different, among them, 19 genes were significant higher in unicellular species (FDR-adjusted *P* < 0.05). When refer to the dS, we found 10 genes were significantly different, among them, 6 genes were significant higher in unicellular species (FDR-adjusted *P* < 0.05). Then we identified 14 putative fast-evolving genes and 11 putative positively selected genes of unicellular species, we analyzed the function of positively selected sites of the overlap genes of putative fast-evolving and positively selected genes, and found some sites were close to the important functional region of the proteins. Photosynthesis is the process to transform and store solar energy by chloroplast, it plays a vital role in the survival of algae, this study is the first to use the chloroplast genomes to analysis the evolutionary relationship between colonial and unicellular species in Chlamydomonadales. We found more genes have higher substitution rates in unicellular species and proposed that the fast-evolving and positively selected two genes, *psbA* and *psbC*, may help to improve the photosynthetic efficiency of unicellular species in Chlamydomonadales.

## Introduction

The volvocine algae belong to Chlamydomonadales (Chlorophyta, Chlorophyceae). This group of algae span the full range of organizational complexity, from unicellular species to colonial species, thus these algae are ideal model organisms to study the fundamental issues related to the transition to multicellularity. In recent years, many chloroplast genomes of Chlamydomonadales species have been sequenced using the application of next generation sequencing technology, this provided massive data for us to study the nucleotide substitution rates based on the chloroplast protein-coding genes data sets (Smith and Lee, 2009; Smith and Lee, 2010; Hamaji et al., 2013; Smith et al., 2013; Del et al., 2015; Lemieux et al., 2015; Featherston et al., 2016; Hu et al., 2019). However, due to the limited number of chloroplast genomes of colonial volvocine algae, little is known about the evolutionary relationship between colonial and unicellular species in Chlamydomonadales based on chloroplast genomes.

The nucleotide substitution rates are often used as the criterion to reflect the selection pressure. The nonsynonymous substitution rates (dN) can cause an amino acid change, the synonymous substitution rates (dS) do not cause an amino acid change. The dN/dS is the ratio of nonsynonymous substitution and synonymous substitution, the ratio of dN/dS is the measure of natural selection acting on the protein. According to Yang (Yang, 2007), dN/dS < 1 means negative purifying selection, dN/dS = 1 means neutral evolution, dN/dS > 1 means positive selection. Generally, the chloroplast protein-coding genes need to maintain the photosynthetic function, so these genes are conserved and have a low dN/dS ratio (Smith, 2015). Meanwhile, a relatively high dN/dS ratio could be interpreted as the positive or relaxed selection (Guisinger et al., 2008), the evolutionary analysis can reveal how species adaptive under selection pressure. For example, Guisinger et al. (2008) calculated the nucleotide substitution rates of chloroplast protein-coding genes of angiosperm, and found unprecedented accumulation of nucleotide substitutions in Geraniaceae, then a model was proposed to illustrate this phenomenon. Zhang et al. (2018) studied the molecular evolution of chloroplast protein-coding genes of an Antarctic sea ice alga *Chlamydomonas* sp. and revealed the adaptive mechanism of sed-ice environment.

In this study, we determined six chloroplast genomes of colonial volvocine algae, these genomes provide valuable opportunity for us to conduct the evolutionary study in Chlamydomonadales. Based on the chloroplast protein-coding genes, we examined the nucleotide substitution rates of Chlamydomonadales species and found that more genes have higher substitution rates in unicellular species when compared with colonial species. Then we identified the putative fast-evolving genes and positively selected genes of unicellular species, based on our analysis, we proposed that the positively selected sites of specific chloroplast protein-coding genes may improve the photosystem efficiency, and our photosynthetic experiment further support our conclusion.

## Materials and Methods

### Sampling, Culture Conditions, DNA Extraction, and Species Identification

The strains described in this study were isolated from water samples and deposited in the Freshwater Algae Culture Collection at the Institute of Hydrobiology (FACHB collection), Wuhan, Hubei Province, China. The *Colemanosphaera charkowiensis* (strain FACHB-2326) were collected from a river in Weihe (36°19′7″ N, 119°28′54″ E), Weifang, Shandong Province, China, in September 2017. The *Volvulina compacta* (strain FACHB-2337) were collected from a river in Tangxihe (31°7′13″ N, 108°49′10″ E), Chongqing, China, in June 2017. The *Pandorina colemaniae* (strain FACHB-2361) were collected from a river in Wuhe (36°22′25″ N, 119°24′52″ E), Weifang, Shandong Province, China, in September 2017. The *Pandorina morum* (strain FACHB-2362) were collected from a river in Weihe (36°8′1″ N, 119°25′59″ E), Weifang, Shandong Province, China, in September 2017. The *Colemanosphaera angeleri* (strain FACHB-2363) were collected from a river in Weihe (36°30′2″ N, 119°24′44″ E), Weifang, Shandong Province, China, in September 2017. The *Yamagishiella unicocca* (strain FACHB-2364) were collected from a pool in Dichi (47°18′20″ N, 120°28′38″ E), Aershan, Inner Mongolia, China, in August 2017. The strains were grown in a conical flask containing artificial freshwater-6 (AF-6) (Kato, 1982) at 20–25°C under a 14 h light: 10 h dark schedule under cool-white fluorescent lamps at an intensity of 1000–2000 lux. Total genomic DNA was extracted using a Universal DNA Isolation Kit (AxyPrep, Suzhou, China) following the manufacturer’s instructions. Species identification was based on morphological observation and phylogenetic analysis based on five chloroplast genes (*rbcL*, *atpB*, *psaA*, *psaB*, and *psbC*), and the sequence data of related species were chosen according to Nozaki et al. (2014). The sequence matrix was aligned by MAFFT v7.394 (Katoh and Standley, 2013), and the ambiguously aligned regions were further manually edited and adjusted by eye using MEGA7 (Kumar et al., 2016). jModelTest v.2.1.7 (Darriba et al., 2012) was used to determine the evolutionary model, which was then analyzed using Bayesian inference (BI) with MrBayes v3.2.6 (Ronquist et al., 2012) and maximum likelihood (ML) with RAxML v8.2.10 (Stamatakis, 2014). Microphotographs were taken by using an Olympus BX53 (Tokyo, Japan) light microscope with an Olympus DP80 digital camera and cellSens standard image analysis software (Tokyo, Japan).

### Library Preparation, Sequencing, Genome Assembly, and Annotation

A sequencing library was prepared using an NEBNext Ultra DNA Library Prep Kit for Illumina (New England Biolabs, United States) and sequenced with an Illumina NovaSeq6000 at Novogene (Beijing, China). The data were trimmed using SOAPnuke v1.3.0 (Chen et al., 2017) and then assembled with SPAdes v3.10.1 (Bankevich et al., 2012). The resulting assembly contigs were determined to be form the chloroplast genome based on the following criteria: (1) blast searches of publicly available chloroplast genomes of Chlorophyta algae species with significant e-values (1e-5); (2) the GC content of the contig is less than 45% (the GC content of green algae chloroplast genomes that have been sequenced to date is normally less than 45%); and (3) the sequencing depth is higher than 100×. Then, the trimmed reads were aligned to the resulting assembly contigs by BWA-MEM v0.7.12 (Li, 2013). If reads mapped two contigs at the same time, we determined the order of contigs, and after confirming the orders of the contigs, the sequence we produced was then rechecked by Sanger dideoxy sequencing technology and synteny analysis with related species. The chloroplast genomes were initially annotated using CpGAVAS (Liu et al., 2012). Protein-coding genes were further polished using Blast with genes from the available colonial volvocine chloroplast genes. All chloroplast genome sequences have been submitted to GenBank, the accession number was listed in Table 1.

**TABLE 1:** GenBank accession numbers of Chlamydomonadales species used in this study.

**Species**	**Accession number**	**Species**	**Accession number**
*Carteria cerasiformis*	KT625420	*Eudorina elegans*	MH161344
*Carteria sp.*	KT625419	*Gonium pectorale*	AP012494
*Characiochloris acuminata*	KT625418	*Haematococcus lacustris*	KT625205 – KT625298
*Chlamydomonas applanata*	KT625417	*Lobochlamys culleus*	KT625151 – KT625204
*Chlamydomonas asymmetrica*	KT624933 – KT625007	*Lobochlamys segnis*	KT624806 – KT624869
*Chlamydomonas leiostraca*	KX828176	*Oogamochlamys gigantea*	KT625412
*Chlamydomonas reinhardtii*	NC_005353	***Pandorina colemaniae***	**MH511720 – MH511732**
*Chlorogonium capillatum*	KT625085 – KT625091	***Pandorina morum***	**MH511697 – MH511719**
*Chloromonas perforata*	KT625416	*Phacotus lenticularis*	KT625422
*Chloromonas radiata*	KT625008 – KT625084	*Pleodorina starrii*	JX977846
***Colemanosphaera angeleri***	**MH511734**	*Tetrabaena socialis*	KX232643
***Colemanosphaera charkowiensis***	**MH511733**	***Volvulina compacta***	**MH511735 – MH511763**
*Dunaliella salina*	GQ250046	*Volvox carteri f. nagariensis*	GU084820
*Eudorina cylindrica*	MH161345	***Yamagishiella unicocca***	**MH511764 – MH511802**

*Bold indicates chloroplast genome sequenced in this study.*

### Phylogenomic Analysis

Our study aims to reveal the evolutionary relationship between colonial and unicellular species in Chlamydomonadales based on the protein-coding genes of chloroplast genomes, we tried to ensure the gene we analyzed are exist in all species, however, we found some species may have limited number of protein-coding genes and some genes of specific species have poor alignment with other species, to keep a balance between the number of genes and the number of species, 12 colonial species and 16 unicellular species were chosen and species information was listed in Table 1. The data set was assembled from the following 55 protein-coding genes: *atpA*, *atpB*, *atpE*, *atpF*, *atpH*, *atpI*, *ccsA*, *cemA*, *chlB*, *chlL*, *chlN*, *clpP*, *petA*, *petB*, *petD*, *petG*, *petL*, *psaB*, *psaC*, *psaJ*, *psbA*, *psbB*, *psbC*, *psbD*, *psbE*, *psbF*, *psbH*, *psbI*, *psbK*, *psbL*, *psbM*, *psbN*, *psbT*, *psbZ*, *rbcL*, *rpl14*, *rpl16*, *rpl2*, *rpl20*, *rpl23*, *rpl36*, *rpl5*, *rps11*, *rps12*, *rps14*, *rps18*, *rps19*, *rps2*, *rps3*, *rps4*, *rps7*, *rps8*, *rps9*, *tufa*, and *ycf4*. The method of phylogenomic analysis was mainly refer to Lemieux et al. (2015). All genes were aligned using MUSCLE v3.8 (Edgar, 2004), and the alignments of all genes were converted into a codon alignment by TranslatorX (Abascal et al., 2010). The ambiguously aligned regions in alignment were excluded using Gblocks0.91b (Castresana, 2000) with the options −t = c, −b3 = 5, −b4 = 5, and −b5 = half. All alignments were concatenated using Phyutility v2.2.6 (Smith and Dunn, 2008), and then the Degen1.pl 1.2 script (Regier et al., 2010) was applied to the concatenated alignment. jModeltest v.2.1.7 (Darriba et al., 2012) was used to determine the evolutionary model. The data was partitioned by gene, with the model applied to each partition. Phylogenies were inferred using ML and BI methods. ML analyses were carried out using RAxML v8.2.10 (Stamatakis, 2014) and the GTRGAMMA model of sequence evolution. Bootstrap analysis with 1,000 replicates of the dataset for ML was performed to estimate statistical reliability. Bayesian analyses were performed with MrBayes v3.2.6 (Ronquist et al., 2012) with the GTR + I + G model. Markov chain Monte Carlo (MCMC) analyses were run with four Markov chains (three heated, one cold) for 1,000,000 generations, with trees sampled every 500 generations. Each time the diagnostics were calculated, a fixed proportion of samples (burninfrac = 0.25) were discarded from the beginning of the chain. A stationary distribution was assumed when the average standard deviation of the split frequencies was lower than 0.01.

### Evolutionary Analysis

The CODEML program of PAML v4.9 (Yang, 2007) with the ML model (runmode = −2, CodonFreq = 2) was used to measure the values of dS and dN, the analysis was based on 55 chloroplast protein-coding genes. As *Chloromonas radiata* belongs, with *Carteria cerasiformis* and *Carteria* sp., to a clade that is sister to all the other species, so *C. radiata* was used as reference. Comparisons of the evolutionary rates were conducted using the two-tailed Wilcoxon rank sum test. The multiple testing was corrected by applying the false discovery rate method (FDR) (Benjamini and Hochberg, 1995) as implemented in R.^1^ The phylogenetic tree was used as a constraint tree, but branch lengths were inferred by using PAML.

The ML method is a pairwise approach to estimate the dN/dS ratio, a dN/dS ratio may indicate in one or both species, and some specific sites under positive selection may remain undetected (Dussert et al., 2018). So, two precise assessments were used to detected the difference of dN/dS and positive selection.

We use the branch model to test whether unicellular species have a different dN/dS ratio relative to the colonial species. The unicellular species were labeled as the foreground branch. A null model (model = 0), where one dN/dS ratio was fixed across all species, was compared with an alternative model (model = 2), where the unicellular species was allowed to have a different dN/dS. Likelihood ratio tests (LRT) were used to test model fit and the Chi-square test was applied for testing *P* values. The multiple testing was corrected by FDR. Genes were considered putative fast-evolving genes if they had an FDR-adjusted *P* < 0.05 and a higher dN/dS ratio in the foreground branch than in the background branches.

We use the branch-site model to find genes that potentially experienced positive selection. The improved branch-site model (model = 2, Nsites = 2) was used to detect signatures of positive selection on individual codons in a specific branch (Zhang et al., 2005). The unicellular species were set as the foreground branch. The null model assumed no positive selection occurred on the foreground branch (fix_omega = 1, omega = 1), and the alternative model assumed that sites on the foreground branch were under positive selection (fix_omega = 0, omega = 2). LRT were used to test model fit and the Chi-square test was applied for testing *P* values. We performed a correction for multiple testing using an FDR criterion, and Bayes empirical Bayes (BEB) method was used to statistically identify sites under positive selection. Genes were considered putative selected genes if they had an FDR-adjusted *P* < 0.05.

The overlap gene represent a group of genes that are not only putative fast-evolving genes but also putative positive selected genes, we analyzed the function of positive selected sites of overlap genes, the functional information were derived from the Uniprot.^2^ The three-dimensional (3D) structures were predicted using Phyre2 (Kelley et al., 2015). The 3D structures were visualized by ePlant Web server (Fucile et al., 2011).

## Results

### Species Identification

**FIGURE 1:**
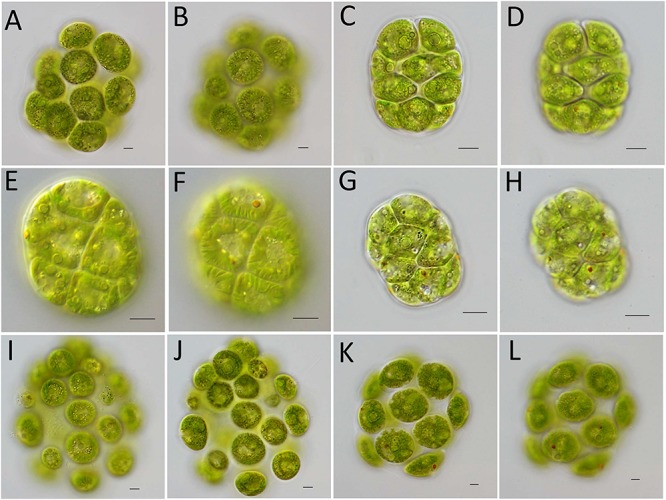
Light microscopy of vegetative colonies of six species of colonial volvocine algae. **(A,B)** *Colemanosphaera charkowiensis*, strain FACHB-2326. **(A)** The chloroplast had more than two pyrenoids of almost identical size. **(B)** Only two contractile vacuoles distributed near the base of the flagella, the chloroplast had prominent longitudinal striations. **(C,D)** *Volvulina compacta*, strain FACHB-2337. **(C)** Colony ellipsoidal in shape, cells were embedded in a gelatinous matrix formed a hollow sphere. **(D)** Cells were more or less contiguous and appeared nearly tetragonal by mutual compression. **(E,F)** *Pandorina colemaniae*, strain FACHB-2361. **(E)** The chloroplast had more than two pyrenoids. **(F)** Colony were ellipsoidal in shape and contained 8 cells compactly arranged in a gelatinous matrix. **(G,H)** *Pandorina morum*, strain FACHB-2362. **(G)** The chloroplast contained a single, basal pyrenoid. **(H)** Colony were ellipsoidal in shape and contained 8 cells compactly arranged in a gelatinous matrix. **(I,J)** *Colemanosphaera angeleri*, strain FACHB-2363. **(I)** Only two contractile vacuoles distributed near the base of the flagella. **(J)** The chloroplast contained a large basal pyrenoid and small pyrenoids. **(K,L)** *Yamagishiella unicocca*, strain FACHB-2364. **(K)** The chloroplast only contained a single, basal pyrenoid. **(L)** Only two contractile vacuoles distributed near the base of the flagella. Scale bars: 10 μm.

Vegetative colonies of strain FACHB-2326 were ellipsoidal in shape, composed by 16 cells of approximately identical sizes embedded by gelatinous matrix forming a hollow colonial structure. Cells spherical, the chloroplast contained more than two pyrenoids of almost identical size (Figure 1A) and prominent longitudinal striations (Figure 1B). Only two contractile vacuoles distributed near the base of the flagella (Figure 1B). Based on the morphology of colony, the number and size of pyrenoids, the number of contractile vacuoles, we identified strain FACHB-2326 as *C. charkowiensis*. Vegetative colonies of strain FACHB-2337 were ellipsoidal in shape, cells were embedded in a gelatinous matrix forming a hollow sphere. Cells were more or less contiguous and appeared nearly tetragonal by mutual compression. There were no prominent spaces between adjoining cells. Based on the spaces between adjoining cells and the shape of cells, we identified strain FACHB-2337 as *V. compacta* (Figures 1C,D). Vegetative colonies of strain FACHB-2361 were ellipsoidal in shape and contained eight cells compactly arranged in a gelatinous matrix. The chloroplast had more than two pyrenoids. Based on the shape of cells and the number of pyrenoids, we identified strain FACHB-2361 as *P. colemaniae* (Figures 1E,F). Vegetative colonies of strain FACHB-2362 resembled strain FACHB-2361, but the chloroplast contained a single, basal pyrenoid, so we can identify strain FACHB-2362 as *P. morum* (Figures 1G,H). Vegetative colonies of strain FACHB-2363 resembled strain FACHB-2326, but the chloroplast striations were not prominent and contained a large basal pyrenoid and small pyrenoids, so we identified strain FACHB-2363 as *C. angeleri* (Figures 1I,J). Vegetative colonies of strain FACHB-2364 resembled strain FACHB-2363, but the chloroplast only contained a single, basal pyrenoid, so we identified strain FACHB-2364 as *Y. unicocca* (Figures 1K,L).

**FIGURE 2:**
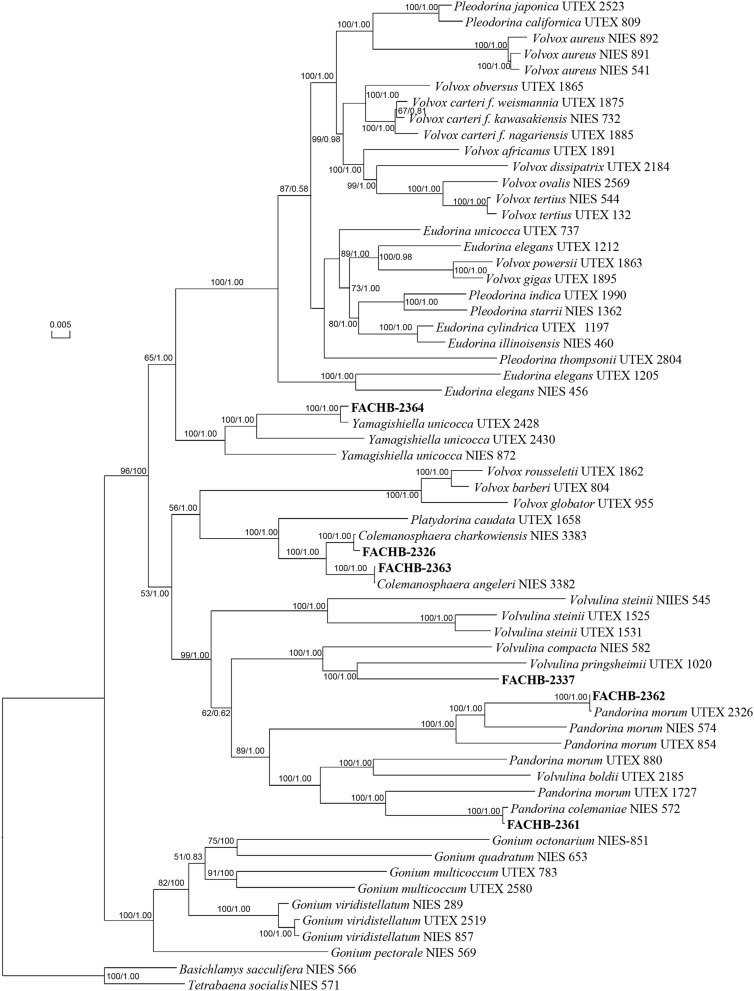
Phylogenetic tree of the colonial volvocine algae based on five chloroplast genes. Numbers on the left and right side at the branches represent bootstrap values and Bayesian posterior probabilities, respectively. Scale bar indicates substitutions per site. Our strains were shown in bold.

The phylogenetic tree based on five genes (Figure 2) supported our morphological identification with high bootstrap value (both 100) and Bayesian posterior probability (both 1.00), except the phylogenetic position of strain FACHB-2337. We noticed that strain FACHB-2337 clustered with *Volvulina pringsheimii* form a lineage. The cells of *V. pringsheimii* are multiangular or circular and not contiguous in surface view, and the colony of *V. pringsheimii* is a hollow sphere more resembles with *Eudorina*. But the colony of strain FACHB-2337 was more compact and resembled with *Pandorina*, and the cells were contiguous in surface view, the morphological observation strongly supported strain FACHB-2337 as *V. compacta* rather than *V. pringsheimii* (Starr, 1962; Nozaki and Kuroiwa, 1990). So, we still considered strain FACHB-2337 as *V. compacta*. In our phylogenetic tree, the phylogenetic position of most species was consisting with the study of Nozaki et al. (2014), except *Volvulina steinii*. The bootstrap value and posterior probability of *V. steinii* clade were relatively low in the study of Nozaki et al. (2014), we found the phylogenetic position of *V. steinii* in our study was consisting with Nakada et al. (2010), and both studies showed high bootstrap value and posterior probability of *V. steinii* clade. Meanwhile, recent study both show polyphyletic of genera *Pandorina* and *Volvulina* (Coleman, 2001; Nakada et al., 2010; Nozaki et al., 2014), so the phylogenetic position of these species may need further study.

### Phylogenomic Analysis and Evolutionary Rate Estimation

**FIGURE 3:**
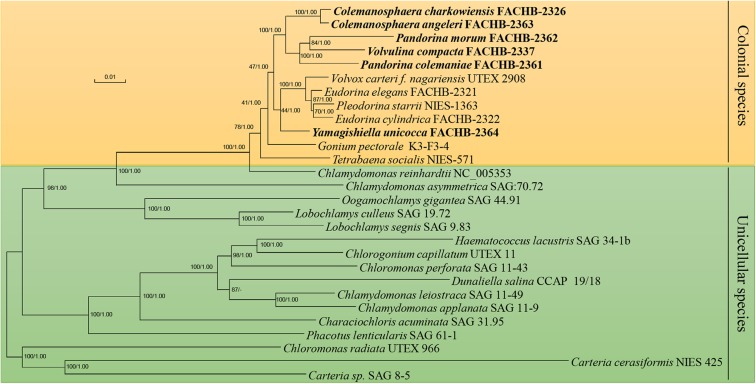
Phylogenetic tree of the Chlamydomonadales species based on the 55 chloroplast genes. Numbers on the left and right side at the branches represent bootstrap values and Bayesian posterior probabilities, respectively. Scale bar indicates substitutions per site. Our strains were shown in bold. The orange background indicated the colonial volvocine algae, and the green background indicated the unicellular species.

We conducted our phylogenomic analysis based on the nucleotide sequence of 55 chloroplast protein-coding genes, ML was carried out using RAxML, Bayesian analyses was performed with MrBayes, the phylogenetic position of most species inferred from both methods are the same except *Dunaliella salina*. The phylogenetic position of *D. salina* inferred from both method have high bootstrap value (87) and posterior probability (1.00), but the result of ML method was in accordance with previous study (Lemieux et al., 2015), so the ML tree was used to represent the result (Figure 3). The phylogenetic position of other unicellular species were consistent with previous study with high bootstrap values and posterior probability values (Yumoto et al., 2013; Lemieux et al., 2015). The phylogenetic position of most colonial species were consistent with previous study (Nozaki et al., 2014) except *P. morum*, we noticed that the *P. morum* together with *V. compacta* formed a lineage instead of *P. colemaniae*, this situation have been reported before (Coleman, 2001), this may mainly due to the polyphyletic of *Pandorina* (Herron, 2016). In our study, this tree was used as the constraint tree for our evolutionary analysis.

Based on the ML method of 55 chloroplast protein-coding genes, the value of dN and dS were compared between colonial and unicellular species in Chlamydomonadales (Table 2 and Supplementary Table S1). When refer to dN, 27 genes were significantly different between the two group of algae, among these genes, we found 19 genes significantly higher in unicellular species. When refer to dS, 10 genes were significantly different between the two group of algae, among these genes, we found six genes significantly higher in unicellular species. Among genes with statistical significance, both comparisons show more genes have higher substitution rates in the unicellular species.

**TABLE 2:** Substitution rates in the chloroplast protein coding genes of Chlamydomonadales species.

**Genes**	**Nonsynonymous substitution rate (dN)**				**Genes**	**Synonymous substitution rate (dS)**			
	**Colonial species**	**Unicellular species**	***P* value**	**FDR**		**Colonial species**	**Unicellular species**	***P* value**	**FDR**
**atpA**	0.0538	0.0708	0.0002	**0.0019**	atpA	2.5084	2.8437	0.1796	0.2994
**atpB**	0.0599	0.0680	0.0037	**0.0127**	atpB	4.0681	4.5126	0.2319	0.3447
atpE	0.0484	0.0604	0.2509	0.3632	atpE	6.2063	4.3520	0.0180	0.0760
**atpF**	0.1053	0.1242	0.0204	**0.0432**	atpF	7.4871	16.4473	0.2319	0.3447
atpH	0.0034	0.0082	0.3649	0.5146	atpH	3.0643	2.6771	0.2134	0.3353
**atpI**	0.0545	0.0646	0.0090	**0.0236**	atpI	3.4857	11.5545	0.5101	0.6376
ccsA	0.1377	0.1258	0.0233	**0.0474**	**ccsA**	4.2649	17.4465	0.0043	**0.0263**
cemA	0.0865	0.0833	0.0749	0.1373	cemA	86.6094	71.0912	0.0749	0.1471
chlB	0.0713	0.0684	0.9417	0.9417	chlB	13.3912	9.2959	0.0429	0.1072
chlL	0.0296	0.0290	0.9029	0.9196	chlL	29.6557	15.0345	0.0068	**0.0372**
chlN	0.0631	0.0649	0.2318	0.3446	chlN	10.6520	36.5932	0.0673	0.1423
clpP	0.0983	0.1044	0.9029	0.9196	clpP	19.8988	31.6639	0.1960	0.3170
petA	0.0884	0.0872	0.6783	0.7937	petA	4.2592	4.2844	0.9417	0.9772
petB	0.0310	0.0329	0.5578	0.7135	petB	4.5533	15.1413	0.0205	0.0804
petD	0.0474	0.0413	0.0429	0.0813	petD	20.9651	5.3063	0.0043	**0.0263**
petG	0.0249	0.0144	0.0057	**0.0173**	petG	2.0122	4.3092	0.0381	0.0998
**petL**	0.0693	0.0976	0.0050	**0.0162**	petL	5.7435	31.2154	0.0381	0.0998
**psaB**	0.0389	0.0431	0.0111	**0.0279**	psaB	2.1499	2.2089	0.4208	0.5644
psaC	0.0167	0.0231	0.0297	0.0582	psaC	3.0107	2.3111	0.0137	0.0629
psaJ	0.1962	0.1682	0.0012	**0.0054**	psaJ	89.4841	79.4622	0.0120	0.0599
psbA	0.0193	0.0172	0.2040	0.3117	psbA	0.9338	0.8313	0.0338	0.0998
**psbB**	0.0238	0.0311	0.0003	**0.0019**	psbB	2.8980	2.3860	0.0381	0.0998
**psbC**	0.0217	0.0309	0.0000	**0.0007**	**psbC**	1.7756	2.5923	0.0016	**0.0181**
**psbD**	0.0091	0.0177	0.0004	**0.0022**	psbD	1.8545	1.8957	0.9805	0.9805
psbE	0.0365	0.0385	0.7883	0.8502	psbE	2.5159	2.5457	0.6430	0.7688
psbF	0.0901	0.0718	0.0827	0.1431	psbF	2.4090	4.3547	0.0749	0.1471
psbH	0.1068	0.1097	0.3931	0.5273	psbH	4.1929	4.6759	0.8262	0.9088
**psbI**	0.0248	0.0431	0.0002	**0.0019**	psbI	2.4077	10.9549	0.0539	0.1235
psbK	0.0704	0.0685	0.6962	0.7978	psbK	19.9463	16.8952	0.2515	0.3640
psbL	0.0026	0.0086	0.8255	0.8732	psbL	2.6263	3.9338	0.0337	0.0998
psbM	0.0701	0.0666	0.6254	0.7817	psbM	58.1957	42.9158	0.1643	0.2824
**psbN**	0.0356	0.0489	0.0029	**0.0107**	psbN	31.0494	32.1522	0.7144	0.8186
**psbT**	0.0442	0.0527	0.0156	**0.0358**	psbT	2.2940	5.6240	0.0264	0.0906
psbZ	0.0897	0.0754	0.0063	**0.0181**	psbZ	7.7164	5.4737	0.0603	0.1327
**rbcL**	0.0329	0.0370	0.0013	**0.0054**	**rbcL**	1.1745	1.4900	0.0008	**0.0136**
rpl14	0.0607	0.0600	0.5097	0.6674	rpl14	60.8149	30.8589	0.0481	0.1151
rpl16	0.0713	0.0770	0.7696	0.8465	rpl16	2.9936	5.5601	0.5101	0.6376
**rpl2**	0.0746	0.0906	0.0002	**0.0019**	rpl2	3.2843	3.4862	0.4208	0.5644
**rpl20**	0.0803	0.1081	0.0003	**0.0019**	rpl20	3.6333	3.8184	0.5747	0.7024
**rpl23**	0.1301	0.1642	0.0008	**0.0042**	rpl23	6.8021	15.0313	0.7884	0.8850
rpl36	0.0936	0.0672	0.0025	**0.0097**	rpl36	19.1638	5.8651	0.0001	**0.0070**
rpl5	0.0882	0.0766	0.0003	**0.0020**	rpl5	7.9760	4.8853	0.0003	**0.0092**
rps11	0.0682	0.0623	0.1796	0.2822	rps11	18.1354	36.6681	0.1367	0.2425
rps12	0.0339	0.0358	0.3790	0.5211	**rps12**	3.0276	6.9315	0.0023	**0.0210**
**rps14**	0.0518	0.0849	0.0000	**0.0010**	rps14	2.3847	2.6426	0.5101	0.6376
**rps18**	0.0918	0.1012	0.0179	**0.0395**	rps18	4.5287	37.9676	0.9417	0.9772
rps19	0.0770	0.0775	0.6430	0.7859	rps19	4.1834	4.0922	0.3932	0.5544
**rps2**	0.2542	0.2932	0.0001	**0.0010**	rps2	13.4798	20.1601	0.9805	0.9805
rps3	0.2526	0.2256	0.0084	**0.0231**	rps3	11.1442	16.8238	0.0233	0.0853
rps4	0.1034	0.0966	0.0120	**0.0286**	rps4	8.0146	18.3266	0.9417	0.9772
rps7	0.0904	0.0891	0.6783	0.7937	rps7	5.3497	24.4733	0.1367	0.2425
rps8	0.1406	0.1502	0.1643	0.2658	**rps8**	3.9437	20.9626	0.0027	**0.0212**
rps9	0.1418	0.1312	0.1243	0.2071	rps9	7.9183	48.1431	0.6783	0.7938
tufA	0.0631	0.0626	0.7144	0.8018	**tufA**	3.0984	4.2509	0.0010	**0.0136**
ycf4	0.1102	0.1262	0.0832	0.1431	ycf4	5.0050	31.1855	0.0923	0.1750

*Genes of substitution rates significantly higher in unicellular species and FDR less than 0.05 were showed in bold.*

**FIGURE 4:**
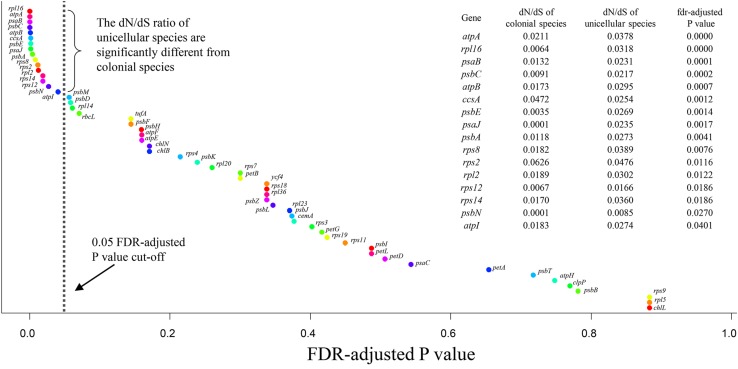
Plot showing ranked FDR-adjusted *P* values for 55 chloroplast protein-coding genes. *P* value were obtained from the branch model likelihood ratio tests, were applied by the false discovery rate method.

The branch model was used to compare the dN/dS ratio between colonial and unicellular species based on the chloroplast protein-coding genes, the LRT was used to compare the fit of two models. The null model (H0) assumed that all tree branches evolved at the same rate (the same dN/dS ratio), the alternative model assumed that the foreground branch (the unicellular species) could evolved at a different rate (different dN/dS ratio). We found the FDR-adjusted *P* value of 16 genes were less than 0.05, this indicates the dN/dS ratio of these genes were significantly different among the unicellular and colonial species (Figure 4 and Supplementary Table S2). Among these genes, the dN/dS ratio of 14 genes (*atpA*, *rpl16*, *psaB*, *psbC*, *atpB*, *psbE*, *psaJ*, *psbA*, *rps8*, *rpl2*, *rps12*, *rps14*, *psbN*, *atpI*) were higher in the unicellular species compared with the colonial species, so these genes were considered putative fast-evolving genes.

**FIGURE 5:**
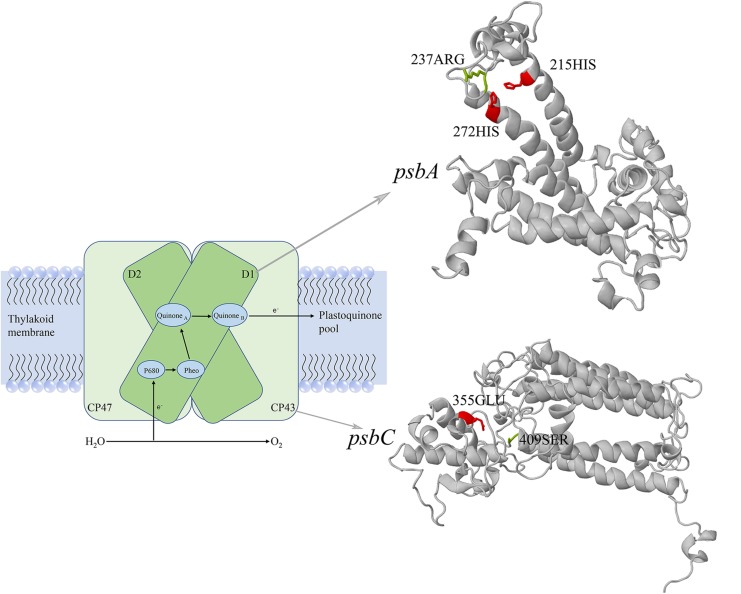
The three-dimensional structures of *psbA* and *psbC*. The *psbA* encodes photosystem II reaction center protein D1, the *psbC* encodes photosystem II CP43 chlorophyll apoprotein. The positively selected sites were showed in green, and the functional sites of *Chlamydomonas reinhardtii* were showed in red. The schematic model of photosystem II was drawn with reference from Yamamoto (2001).

The positive selection analysis was performed based on the branch-site model, and we also conducted the comparison between the null and alternative models. The null model considered the foreground branch only have dN/dS = 1, and the alternative model considered sites on the foreground branch have dN/dS > 1 (positive selection). We used the Chi-square test to testing *P* values, after the FDR correction, we found 11 genes (*psaB*, *psbB*, *psbC*, *rbcL*, *tufA*, *psbA*, *rps4*, *rpl5*, *rpl16*, *rps12*, *atpF*) have the FDR-adjusted *P* value lower than 0.05 (Supplementary Table S3), and we considered these genes as the putative positively selected genes. The overlapped genes between the putative fast-evolving genes and positively selected genes were *psaB*, *psbC*, *psbA*, *rpl16*, and *rps12*. Based on the BEB method, the positively selected sites for each gene were shown in Table 3. We found the *psaB*, *psbC* and *psbA* have sites may likely under positive selection. For *psaB*, 134GLN have posterior probability higher than 95%, 253GLN have posterior probability higher than 90%, but there is no related functional sites information of *Chlamydomonas reinhardtii* in Uniprot, so the positively selected sites of *psaB* reminds further study. For *psbA*, site 237ARG (posterior probability higher than 90%) was close to the 215HIS and 272HIS of *C. reinhardtii*, the 215HIS is the metal binding site of iron and binding site of Quinone (B), the 272HIS is also the metal binding site of iron. For *psbC*, site 409SER (posterior probability higher than 95%) was close to the 355GLU of *C. reinhardtii* which was the metal binding site of calcium-manganese-oxide (Figure 5).

**TABLE 3:** Positively selected sites in overlapped genes of unicellular species.

**Gene**	**lnL H0**	**lnL HA**	**df**	**lnL 2 × | (HA-H0)|**	***P* value**	**FDR**	**Positive selected sites under BEB analysis**
*psaB*	–18601.6212	–18660.9855	1	118.7287	0.0000	0.0000	134Q-0.994, 253Q-0.903, 483T-0.782, 485A-0.821
*psbC*	–10162.7833	–10208.4296	1	91.2926	0.0000	0.0000	155I-0.617, 213C-0.793, 232E-0.887, 366N-0.577, 409S-0.987
*psbA*	–7022.2349	–7038.1916	1	31.9134	0.0000	0.0000	78T-0.627, 237R-0.931, 289L-0.662
*rpl16*	–3671.6974	–3679.2970	1	15.1992	0.0001	0.0006	None
*rps12*	–3430.3255	–3437.4909	1	14.3308	0.0002	0.0008	None

*Number behind hyphen is the posterior probability of BEB analysis.*

## Discussion

In this study, we determined six chloroplast genomes of colonial volvocine algae, this provided opportunity for us to reveal the different evolutionary rate between unicellular and colonial species in Chlamydomonadales. Our analysis was based on the protein-coding genes of 12 colonial volvocine algae and 16 unicellular species, we used the ML method to calculate the value of dN and dS of each gene and each species. The rate of synonymous substitutions and nonsynonymous substitutions of more genes were higher in unicellular species; the nonsynonymous substitution can modify the produced amino acid sequence, among the 27 significantly different genes, we found the nonsynonymous substitution of 19 genes were significantly higher (FDR-adjusted *P* < 0.05) in unicellular species than colonial species. More genes also have higher synonymous substitutions in the unicellular species (6 gene higher in unicellular species among 10 significantly different). All this analysis indicated more genes have higher substitution rates in unicellular species.

Guisinger et al. (2008) have found the increased substitution rates in Geraniaceae, and they proposed that the mutations in chloroplast-targeted genes could leading to increased substitution rates in chloroplast genes. Such explanation would expect rate increased for all chloroplast genes (Wang et al., 2015), since we observed the increased of dN and dS in limited number of genes in this study, the plastid DNA repair mechanism could only partly be one of the reasons responsible for the higher substitution rates in unicellular species. Shen et al. (2009) found higher substitution rates in weakly locomotive species when compared with strongly locomotive species, they associated such phenomenon with the different demand for energy. Likewise, based on our photosynthetic experiment (Supplementary Tables S4, S5), we found that the unicellular species may have lower demand for light when compared with colonial species, here, we speculate that the lower demand for light could indicate a relaxation of constraint on unicellular chloroplast genes compared with colonial species (Björnerfeldt et al., 2006), and the relaxation of constraint allow for more substitutions in the chloroplast genes of unicellular species.

To explore the substitution happens in the unicellular species whether harboring an advantageous that increased individual adaptability, we used the branch model to test whether genes were under fast-evolving in unicellular species, and we used the branch-site model to test whether sites on the unicellular branch were under positive selection. Based on our analysis, 14 genes were considered as the putative fast-evolving genes and 11 genes were considered as the putative positively selected genes, five genes were overlapped among these two group of genes. The overlap genes have higher dN/dS ratio in unicellular species (fast-evolving), meanwhile, they were undergone adaptive molecular changes (positively selection), so the positively selected sites of overlap genes may closely relate to the adaptive of unicellular species. Among the five overlap genes, we analyzed the positively selected sites for each gene by refer to the functional sites of *C. reinhardtii* in Uniprot, and we found two genes (*psbA*, *psbC*) may play an import role in adaption. The *psbA* encodes photosystem II reaction center protein D1, it is one of the two reaction center proteins of photosystem II, the function of this protein is associated with the electron transfer. The *psbC* encodes photosystem II CP43 chlorophyll apoprotein, it is one of the components of the core complex of photosystem II, it binds chlorophyll and helps catalyze the primary light-induced photochemical processes of photosystem II. According to the sites information in Uniprot, the positive selected sites of *psbA* and *psbC* gene were close to the functional sites of the homologous protein in *C. reinhardtii*. One positively selected site of *psbA* was close to the iron binding site and Quinone (B) binding site, this is associated with the formation of the iron-quinone complex (Wydrzynski and Satoh, 2005; Umena et al., 2011). One positively selected site of *psbC* was close to the binding site of calcium-manganese-oxide possibly contributed to the oxidation of water (Govindjee et al., 2010; Najafpour, 2011). In general, these two genes were all act an important role in photosynthesis, the molecular evidence show that their substitutions may contributed to the efficiency of photosystem. Our photosynthetic experiment showed the limitation of iron or calcium have lower impact on unicellular species compared with colonial species (Supplementary Tables S4, S5). We speculate that the lower impact could due to the positive selection sites in the *psbA* and *psbC* gene, the positive selection sites help these two gene have a better binding efficiency with iron or calcium, then allow unicellular species could better utilize the trace amount of iron or calcium left in the culture medium than colonial species. Our experiment further supported our conclusion.

Our study is the first determination of the chloroplast genomes of six colonial volvocine algae, by compared with the chloroplast genomes of colonial volvocine algae, we reveled more genes have higher substitution rates in unicellular species of Chlamydomonadales. We identified the fast-evolving and positively selected genes in unicellular species and found the *psbA* and *psbC* might improve the photosynthetic efficiency of unicellular species. This study not only increased the chloroplast genome information of volvocine algae but also provided useful information to understand the evolutionary relationship between unicellular and colonial species in Chlamydomonadales.

## Author Contributions

YH and WX performed the experiments. YH, WX, and HS analyzed and interpreted the data. YH wrote the manuscript. All authors revised, read, and approved the final version of the manuscript.

## Conflict of Interest Statement

The authors declare that the research was conducted in the absence of any commercial or financial relationships that could be construed as a potential conflict of interest.

## Footnotes

1 http://www.R-project.org

2 http://www.uniprot.org/
