# Supplementary material for: Evolutionary Analysis of Unicellular Species in Chlamydomonadales Through Chloroplast Genome Comparison With the Colonial Volvocine Algae
Source: Front Microbiol. 2019 Jun 18;10:1351. doi: 10.3389/fmicb.2019.01351 (PMC6591512; doi:10.3389/fmicb.2019.01351)
Supplement: Supplementary file 1 [file Table_1.DOCX]

Table S1 The dN/dS ratio calculated by ML method.

|  | Colonial species | | | | | | | | | | | | Unicellular species | | | | | | | | | | | | | | |
| --- | --- | --- | --- | --- | --- | --- | --- | --- | --- | --- | --- | --- | --- | --- | --- | --- | --- | --- | --- | --- | --- | --- | --- | --- | --- | --- | --- |
|  | *Colemanosphaera angeleri* | *Yamagishiella unicocca* | *Volvulina compacta* | *Pandorina colemaniae* | *Gonium pectorale* | *Colemanosphaera charkowiensis* | *Eudorina elegans* | *Eudorina cylindrica* | *Pleodorina starrii* | *Tetrabaena socialis* | *Volvox carteri* | *Pandorina morum* | *Dunaliella salina* | *Lobochlamys segnis* | *Chlamydomonas asymmetrica* | *Chlorogonium capillatum* | *Lobochlamys culleus* | *Haematococcus lacustris* | *Oogamochlamys gigantea* | *Chloromonas perforata* | *Chlamydomonas applanata* | *Characiochloris acuminata* | *Carteria sp.* | *Carteria cerasiformis* | *Phacotus lenticularis* | *Chlamydomonas leiostraca* | *Chlamydomonas reinhardtii* |
| *atpA* | 0.0240 | 0.0275 | 0.0202 | 0.0181 | 0.0231 | 0.0216 | 0.0219 | 0.0215 | 0.0189 | 0.0226 | 0.0193 | 0.0218 | 0.0404 | 0.0215 | 0.0245 | 0.0258 | 0.0291 | 0.0239 | 0.0158 | 0.0215 | 0.0288 | 0.0192 | 0.0243 | 0.0280 | 0.0309 | 0.0335 | 0.0186 |
| *atpB* | 0.0129 | 0.0157 | 0.0145 | 0.0130 | 0.0160 | 0.0155 | 0.0153 | 0.0113 | 0.0145 | 0.0184 | 0.0155 | 0.0166 | 0.0155 | 0.0142 | 0.0222 | 0.0171 | 0.0151 | 0.0103 | 0.0140 | 0.0134 | 0.0163 | 0.0137 | 0.0202 | 0.0116 | 0.0170 | 0.0174 | 0.0192 |
| *atpE* | 0.0096 | 0.0065 | 0.0174 | 0.0077 | 0.0103 | 0.0098 | 0.0072 | 0.0044 | 0.0057 | 0.0121 | 0.0056 | 0.0086 | 0.0256 | 0.0129 | 0.0236 | 0.0118 | 0.0142 | 0.0150 | 0.0073 | 0.0167 | 0.0121 | 0.0217 | 0.0113 | 0.0088 | 0.0165 | 0.0249 | 0.0101 |
| *atpF* | 0.0126 | 0.0152 | 0.0133 | 0.0121 | 0.0105 | 0.0143 | 0.0155 | 0.0170 | 0.0152 | 0.0216 | 0.0143 | 0.0125 | 0.0165 | 0.0070 | 0.0156 | 0.0010 | 0.0259 | 0.0132 | 0.0121 | 0.0182 | 0.0135 | 0.0176 | 0.0145 | 0.0308 | 0.0110 | 0.0086 | 0.0137 |
| *atpH* | 0.0010 | 0.0030 | 0.0010 | 0.0010 | 0.0010 | 0.0010 | 0.0010 | 0.0010 | 0.0010 | 0.0010 | 0.0010 | 0.0010 | 0.0010 | 0.0171 | 0.0010 | 0.0010 | 0.0087 | 0.0010 | 0.0019 | 0.0017 | 0.0018 | 0.0010 | 0.0010 | 0.0037 | 0.0032 | 0.0010 | 0.0019 |
| *atpI* | 0.0196 | 0.0131 | 0.0213 | 0.0161 | 0.0186 | 0.0125 | 0.0101 | 0.0182 | 0.0191 | 0.0184 | 0.0159 | 0.0118 | 0.0302 | 0.0183 | 0.0223 | 0.0010 | 0.0162 | 0.0223 | 0.0178 | 0.0099 | 0.0132 | 0.0010 | 0.0155 | 0.0141 | 0.0236 | 0.0268 | 0.0171 |
| *ccsA* | 0.0368 | 0.0450 | 0.0491 | 0.0379 | 0.0418 | 0.0334 | 0.0187 | 0.0237 | 0.0271 | 0.0264 | 0.0386 | 0.0391 | 0.0237 | 0.0126 | 0.0218 | 0.0208 | 0.0077 | 0.0272 | 0.0375 | 0.0180 | 0.0013 | 0.0013 | 0.0252 | 0.0257 | 0.0289 | 0.0179 | 0.0183 |
| *cemA* | 0.0010 | 0.0010 | 0.0010 | 0.0010 | 0.0010 | 0.0010 | 0.0010 | 0.0010 | 0.0010 | 0.0010 | 0.0010 | 0.0010 | 0.0010 | 0.0139 | 0.0010 | 0.0010 | 0.0010 | 0.0010 | 0.0011 | 0.0036 | 0.0020 | 0.0010 | 0.0010 | 0.0012 | 0.0010 | 0.0010 | 0.0010 |
| *chlB* | 0.0069 | 0.0145 | 0.0010 | 0.0125 | 0.0077 | 0.0113 | 0.0108 | 0.0126 | 0.0128 | 0.0151 | 0.0104 | 0.0114 | 0.0164 | 0.0079 | 0.0166 | 0.0185 | 0.0119 | 0.0117 | 0.0134 | 0.0162 | 0.0149 | 0.0119 | 0.0108 | 0.0010 | 0.0106 | 0.0175 | 0.0128 |
| *chlL* | 0.0010 | 0.0010 | 0.0010 | 0.0010 | 0.0010 | 0.0010 | 0.0010 | 0.0010 | 0.0010 | 0.0010 | 0.0010 | 0.0010 | 0.0010 | 0.0028 | 0.0030 | 0.0085 | 0.0010 | 0.0064 | 0.0010 | 0.0032 | 0.0036 | 0.0038 | 0.0045 | 0.0010 | 0.0035 | 0.0029 | 0.0015 |
| *chlN* | 0.0067 | 0.0144 | 0.0010 | 0.0092 | 0.0109 | 0.0119 | 0.0095 | 0.0101 | 0.0106 | 0.0160 | 0.0077 | 0.0123 | 0.0114 | 0.0116 | 0.0114 | 0.0115 | 0.0091 | 0.0010 | 0.0010 | 0.0010 | 0.0056 | 0.0010 | 0.0062 | 0.0010 | 0.0010 | 0.0010 | 0.0091 |
| *clpP* | 0.0040 | 0.0094 | 0.0073 | 0.0108 | 0.0058 | 0.0045 | 0.0088 | 0.0093 | 0.0120 | 0.0080 | 0.0094 | 0.0010 | 0.0130 | 0.0230 | 0.0141 | 0.0134 | 0.0122 | 0.0010 | 0.0230 | 0.0061 | 0.0119 | 0.0010 | 0.0147 | 0.0020 | 0.0010 | 0.0101 | 0.0087 |
| *petA* | 0.0216 | 0.0185 | 0.0173 | 0.0148 | 0.0256 | 0.0231 | 0.0222 | 0.0233 | 0.0208 | 0.0232 | 0.0184 | 0.0254 | 0.0246 | 0.0320 | 0.0147 | 0.0234 | 0.0363 | 0.0139 | 0.0174 | 0.0242 | 0.0137 | 0.0320 | 0.0291 | 0.0197 | 0.0208 | 0.0159 | 0.0183 |
| *petB* | 0.0110 | 0.0060 | 0.0079 | 0.0065 | 0.0051 | 0.0058 | 0.0079 | 0.0092 | 0.0062 | 0.0128 | 0.0043 | 0.0071 | 0.0112 | 0.0010 | 0.0042 | 0.0069 | 0.0010 | 0.0010 | 0.0010 | 0.0017 | 0.0063 | 0.0082 | 0.0046 | 0.0038 | 0.0033 | 0.0096 | 0.0068 |
| *petD* | 0.0010 | 0.0160 | 0.0148 | 0.0010 | 0.0010 | 0.0127 | 0.0106 | 0.0131 | 0.0010 | 0.0067 | 0.0109 | 0.0132 | 0.0092 | 0.0155 | 0.0010 | 0.0159 | 0.0180 | 0.0121 | 0.0118 | 0.0172 | 0.0189 | 0.0091 | 0.0212 | 0.0265 | 0.0123 | 0.0114 | 0.0212 |
| *petG* | 0.0075 | 0.0109 | 0.0261 | 0.0109 | 0.0114 | 0.0122 | 0.0118 | 0.0111 | 0.0138 | 0.0109 | 0.0138 | 0.0123 | 0.0033 | 0.0010 | 0.0010 | 0.0070 | 0.0010 | 0.0020 | 0.0060 | 0.0102 | 0.0036 | 0.0073 | 0.0042 | 0.0152 | 0.0010 | 0.0010 | 0.0121 |
| *petL* | 0.0213 | 0.0112 | 0.0122 | 0.0156 | 0.0174 | 0.0216 | 0.0077 | 0.0077 | 0.0100 | 0.0155 | 0.0110 | 0.0128 | 0.0016 | 0.0045 | 0.0041 | 0.0132 | 0.0142 | 0.0086 | 0.0012 | 0.0221 | 0.0010 | 0.0150 | 0.0023 | 0.1036 | 0.0010 | 0.0110 | 0.0091 |
| *psaB* | 0.0163 | 0.0187 | 0.0158 | 0.0171 | 0.0165 | 0.0214 | 0.0168 | 0.0177 | 0.0187 | 0.0207 | 0.0203 | 0.0188 | 0.0178 | 0.0202 | 0.0192 | 0.0230 | 0.0219 | 0.0184 | 0.0183 | 0.0212 | 0.0188 | 0.0230 | 0.0156 | 0.0227 | 0.0166 | 0.0168 | 0.0201 |
| *psaC* | 0.0048 | 0.0046 | 0.0067 | 0.0029 | 0.0064 | 0.0112 | 0.0049 | 0.0032 | 0.0043 | 0.0075 | 0.0063 | 0.0087 | 0.0171 | 0.0078 | 0.0172 | 0.0145 | 0.0065 | 0.0155 | 0.0021 | 0.0190 | 0.0132 | 0.0079 | 0.0026 | 0.0010 | 0.0122 | 0.0114 | 0.0068 |
| *psaJ* | 0.0021 | 0.0021 | 0.0025 | 0.0020 | 0.0022 | 0.0023 | 0.0024 | 0.0022 | 0.0023 | 0.0024 | 0.0020 | 0.0020 | 0.0044 | 0.0022 | 0.0021 | 0.0021 | 0.0025 | 0.0016 | 0.0019 | 0.0018 | 0.0019 | 0.0019 | 0.0021 | 0.0018 | 0.0023 | 0.0019 | 0.0024 |
| *psbA* | 0.0201 | 0.0211 | 0.0164 | 0.0207 | 0.0229 | 0.0214 | 0.0200 | 0.0199 | 0.0213 | 0.0245 | 0.0211 | 0.0200 | 0.0298 | 0.0173 | 0.0194 | 0.0237 | 0.0322 | 0.0170 | 0.0174 | 0.0308 | 0.0211 | 0.0264 | 0.0137 | 0.0133 | 0.0176 | 0.0186 | 0.0223 |
| *psbB* | 0.0118 | 0.0069 | 0.0082 | 0.0096 | 0.0076 | 0.0085 | 0.0064 | 0.0095 | 0.0068 | 0.0081 | 0.0079 | 0.0085 | 0.0214 | 0.0156 | 0.0099 | 0.0158 | 0.0120 | 0.0144 | 0.0119 | 0.0101 | 0.0145 | 0.0141 | 0.0093 | 0.0126 | 0.0119 | 0.0142 | 0.0110 |
| *psbC* | 0.0139 | 0.0099 | 0.0092 | 0.0135 | 0.0140 | 0.0107 | 0.0154 | 0.0133 | 0.0124 | 0.0126 | 0.0131 | 0.0114 | 0.0095 | 0.0097 | 0.0142 | 0.0196 | 0.0094 | 0.0133 | 0.0110 | 0.0168 | 0.0133 | 0.0079 | 0.0176 | 0.0096 | 0.0137 | 0.0094 | 0.0137 |
| *psbD* | 0.0040 | 0.0060 | 0.0048 | 0.0045 | 0.0036 | 0.0039 | 0.0055 | 0.0056 | 0.0048 | 0.0069 | 0.0045 | 0.0054 | 0.0180 | 0.0125 | 0.0109 | 0.0074 | 0.0062 | 0.0082 | 0.0110 | 0.0114 | 0.0077 | 0.0072 | 0.0112 | 0.0045 | 0.0163 | 0.0110 | 0.0040 |
| *psbE* | 0.0184 | 0.0168 | 0.0162 | 0.0122 | 0.0189 | 0.0148 | 0.0130 | 0.0121 | 0.0130 | 0.0182 | 0.0105 | 0.0160 | 0.0129 | 0.0129 | 0.0386 | 0.0144 | 0.0149 | 0.0101 | 0.0072 | 0.0272 | 0.0184 | 0.0059 | 0.0213 | 0.0088 | 0.0265 | 0.0216 | 0.0140 |
| *psbF* | 0.0374 | 0.0706 | 0.0411 | 0.0556 | 0.0411 | 0.0210 | 0.0495 | 0.0557 | 0.0593 | 0.0807 | 0.0495 | 0.0078 | 0.0375 | 0.0072 | 0.0769 | 0.0186 | 0.0174 | 0.0099 | 0.0114 | 0.0388 | 0.0079 | 0.0293 | 0.0256 | 0.0087 | 0.0291 | 0.0090 | 0.1275 |
| *psbH* | 0.0266 | 0.0195 | 0.0327 | 0.0288 | 0.0225 | 0.0210 | 0.0190 | 0.0249 | 0.0221 | 0.0311 | 0.0343 | 0.0381 | 0.0196 | 0.0517 | 0.0234 | 0.0290 | 0.0335 | 0.0103 | 0.0390 | 0.0121 | 0.0247 | 0.0446 | 0.0237 | 0.0238 | 0.0352 | 0.0151 | 0.0314 |
| *psbI* | 0.0104 | 0.0120 | 0.0085 | 0.0093 | 0.0065 | 0.0127 | 0.0079 | 0.0122 | 0.0118 | 0.0136 | 0.0117 | 0.0127 | 0.0051 | 0.0010 | 0.0049 | 0.0081 | 0.0265 | 0.0103 | 0.0364 | 0.0461 | 0.0061 | 0.0056 | 0.0053 | 0.0010 | 0.0149 | 0.0142 | 0.0096 |
| *psbK* | 0.0024 | 0.0184 | 0.0241 | 0.0109 | 0.0164 | 0.0010 | 0.0206 | 0.0093 | 0.0080 | 0.0010 | 0.0195 | 0.0010 | 0.0320 | 0.0067 | 0.0196 | 0.0088 | 0.0155 | 0.0191 | 0.0056 | 0.0204 | 0.0013 | 0.0347 | 0.0422 | 0.0031 | 0.0288 | 0.0012 | 0.0027 |
| *psbL* | 0.0010 | 0.0010 | 0.0010 | 0.0010 | 0.0010 | 0.0010 | 0.0010 | 0.0010 | 0.0010 | 0.0010 | 0.0010 | 0.0010 | 0.0068 | 0.0010 | 0.0107 | 0.0083 | 0.0010 | 0.0094 | 0.0010 | 0.0010 | 0.0010 | 0.0010 | 0.0010 | 0.0010 | 0.0010 | 0.0010 | 0.0010 |
| *psbM* | 0.0010 | 0.0010 | 0.0011 | 0.0055 | 0.0010 | 0.0010 | 0.0010 | 0.0010 | 0.0105 | 0.0011 | 0.0010 | 0.0010 | 0.0199 | 0.0010 | 0.0115 | 0.0011 | 0.0010 | 0.0011 | 0.0010 | 0.0012 | 0.0068 | 0.0010 | 0.0010 | 0.0134 | 0.0011 | 0.0099 | 0.0010 |
| *psbN* | 0.0010 | 0.0010 | 0.0010 | 0.0010 | 0.0010 | 0.0142 | 0.0010 | 0.0010 | 0.0010 | 0.0010 | 0.0010 | 0.0140 | 0.0010 | 0.0205 | 0.0010 | 0.0010 | 0.0010 | 0.0049 | 0.0314 | 0.0010 | 0.0039 | 0.0010 | 0.0117 | 0.0010 | 0.0010 | 0.0154 | 0.0010 |
| *psbT* | 0.0206 | 0.0204 | 0.0161 | 0.0168 | 0.0195 | 0.0379 | 0.0233 | 0.0170 | 0.0168 | 0.0160 | 0.0170 | 0.0231 | 0.0089 | 0.0273 | 0.0074 | 0.0055 | 0.0800 | 0.0146 | 0.0413 | 0.0139 | 0.0128 | 0.0095 | 0.0010 | 0.0046 | 0.0141 | 0.0110 | 0.0325 |
| *psbZ* | 0.0151 | 0.0342 | 0.0036 | 0.0154 | 0.0283 | 0.0054 | 0.0167 | 0.0166 | 0.0170 | 0.0268 | 0.0216 | 0.0057 | 0.0155 | 0.0224 | 0.0264 | 0.0224 | 0.0202 | 0.0157 | 0.0159 | 0.0022 | 0.0183 | 0.0189 | 0.0230 | 0.0112 | 0.0147 | 0.0201 | 0.0377 |
| *rbcL* | 0.0288 | 0.0280 | 0.0314 | 0.0262 | 0.0262 | 0.0282 | 0.0278 | 0.0291 | 0.0251 | 0.0288 | 0.0275 | 0.0292 | 0.0376 | 0.0227 | 0.0247 | 0.0247 | 0.0239 | 0.0149 | 0.0160 | 0.0293 | 0.0307 | 0.0267 | 0.0226 | 0.0267 | 0.0317 | 0.0226 | 0.0369 |
| *rpl14* | 0.0010 | 0.0010 | 0.0010 | 0.0010 | 0.0010 | 0.0010 | 0.0010 | 0.0010 | 0.0010 | 0.0010 | 0.0010 | 0.0010 | 0.0010 | 0.0071 | 0.0051 | 0.0098 | 0.0065 | 0.0010 | 0.0092 | 0.0014 | 0.0092 | 0.0078 | 0.0061 | 0.0010 | 0.0010 | 0.0117 | 0.0010 |
| *rpl16* | 0.0225 | 0.0265 | 0.0275 | 0.0225 | 0.0225 | 0.0229 | 0.0217 | 0.0214 | 0.0209 | 0.0305 | 0.0228 | 0.0275 | 0.0139 | 0.0429 | 0.0302 | 0.0242 | 0.0222 | 0.0140 | 0.0190 | 0.0444 | 0.0028 | 0.0296 | 0.0320 | 0.0268 | 0.0155 | 0.0138 | 0.0205 |
| *rpl2* | 0.0271 | 0.0255 | 0.0261 | 0.0261 | 0.0221 | 0.0186 | 0.0252 | 0.0228 | 0.0234 | 0.0210 | 0.0216 | 0.0175 | 0.0297 | 0.0367 | 0.0278 | 0.0230 | 0.0291 | 0.0322 | 0.0194 | 0.0282 | 0.0239 | 0.0244 | 0.0250 | 0.0299 | 0.0236 | 0.0209 | 0.0212 |
| *rpl20* | 0.0274 | 0.0225 | 0.0211 | 0.0202 | 0.0229 | 0.0194 | 0.0227 | 0.0222 | 0.0225 | 0.0229 | 0.0257 | 0.0183 | 0.0317 | 0.0324 | 0.0373 | 0.0321 | 0.0149 | 0.0389 | 0.0345 | 0.0313 | 0.0321 | 0.0348 | 0.0143 | 0.0370 | 0.0300 | 0.0331 | 0.0202 |
| *rpl23* | 0.0126 | 0.0167 | 0.0225 | 0.0419 | 0.0390 | 0.0182 | 0.0185 | 0.0180 | 0.0200 | 0.0159 | 0.0172 | 0.0152 | 0.0139 | 0.0239 | 0.0391 | 0.0383 | 0.0202 | 0.0308 | 0.0015 | 0.0328 | 0.0340 | 0.0169 | 0.0068 | 0.0195 | 0.0253 | 0.0466 | 0.0198 |
| *rpl36* | 0.0095 | 0.0096 | 0.0010 | 0.0108 | 0.0049 | 0.0077 | 0.0071 | 0.0091 | 0.0055 | 0.0119 | 0.0126 | 0.0080 | 0.0229 | 0.0282 | 0.0012 | 0.0798 | 0.0351 | 0.0961 | 0.0855 | 0.0200 | 0.0477 | 0.0137 | 0.0504 | 0.0287 | 0.0762 | 0.0170 | 0.0135 |
| *rpl5* | 0.0115 | 0.0107 | 0.0113 | 0.0090 | 0.0119 | 0.0097 | 0.0127 | 0.0121 | 0.0114 | 0.0100 | 0.0141 | 0.0095 | 0.0171 | 0.0277 | 0.0155 | 0.0196 | 0.0298 | 0.0110 | 0.0082 | 0.0193 | 0.0137 | 0.0146 | 0.0168 | 0.0183 | 0.0207 | 0.0143 | 0.0148 |
| *rps11* | 0.0093 | 0.0032 | 0.0048 | 0.0050 | 0.0028 | 0.0032 | 0.0029 | 0.0034 | 0.0035 | 0.0036 | 0.0042 | 0.0033 | 0.0010 | 0.0144 | 0.0010 | 0.0010 | 0.0168 | 0.0012 | 0.0366 | 0.0010 | 0.0010 | 0.0010 | 0.0134 | 0.0054 | 0.0010 | 0.0010 | 0.0029 |
| *rps12* | 0.0094 | 0.0094 | 0.0107 | 0.0130 | 0.0138 | 0.0113 | 0.0105 | 0.0114 | 0.0134 | 0.0124 | 0.0100 | 0.0105 | 0.0010 | 0.0050 | 0.0078 | 0.0095 | 0.0065 | 0.0095 | 0.0080 | 0.0125 | 0.0067 | 0.0033 | 0.0071 | 0.0229 | 0.0076 | 0.0073 | 0.0145 |
| *rps14* | 0.0191 | 0.0175 | 0.0180 | 0.0207 | 0.0211 | 0.0234 | 0.0262 | 0.0222 | 0.0260 | 0.0209 | 0.0234 | 0.0240 | 0.0487 | 0.0496 | 0.0551 | 0.0306 | 0.0452 | 0.0259 | 0.0453 | 0.0286 | 0.0148 | 0.0372 | 0.0241 | 0.0401 | 0.0322 | 0.0303 | 0.0220 |
| *rps18* | 0.0234 | 0.0239 | 0.0279 | 0.0249 | 0.0227 | 0.0333 | 0.0189 | 0.0105 | 0.0187 | 0.0224 | 0.0109 | 0.0365 | 0.0352 | 0.0357 | 0.0010 | 0.0339 | 0.0416 | 0.0010 | 0.0281 | 0.0409 | 0.0356 | 0.0140 | 0.0010 | 0.0015 | 0.0010 | 0.0031 | 0.0433 |
| *rps19* | 0.0158 | 0.0153 | 0.0192 | 0.0137 | 0.0199 | 0.0177 | 0.0210 | 0.0185 | 0.0189 | 0.0230 | 0.0218 | 0.0194 | 0.0226 | 0.0205 | 0.0178 | 0.0112 | 0.0469 | 0.0252 | 0.0101 | 0.0336 | 0.0089 | 0.0189 | 0.0241 | 0.0177 | 0.0283 | 0.0250 | 0.0249 |
| *rps2* | 0.0169 | 0.0214 | 0.0153 | 0.0174 | 0.0192 | 0.0190 | 0.0191 | 0.0219 | 0.0228 | 0.0176 | 0.0192 | 0.0192 | 0.0149 | 0.0224 | 0.0206 | 0.0238 | 0.0268 | 0.0024 | 0.0347 | 0.0213 | 0.0232 | 0.0212 | 0.0165 | 0.0288 | 0.0367 | 0.0195 | 0.0187 |
| *rps3* | 0.0286 | 0.0231 | 0.0184 | 0.0318 | 0.0225 | 0.0214 | 0.0203 | 0.0218 | 0.0199 | 0.0259 | 0.0225 | 0.0217 | 0.0243 | 0.0294 | 0.0225 | 0.0231 | 0.0276 | 0.0280 | 0.0289 | 0.0201 | 0.0250 | 0.0337 | 0.0220 | 0.0026 | 0.0242 | 0.0230 | 0.0191 |
| *rps4* | 0.0184 | 0.0196 | 0.0088 | 0.0073 | 0.0128 | 0.0097 | 0.0169 | 0.0191 | 0.0178 | 0.0115 | 0.0173 | 0.0119 | 0.0010 | 0.0167 | 0.0100 | 0.0097 | 0.0133 | 0.0131 | 0.0179 | 0.0010 | 0.0111 | 0.0242 | 0.0095 | 0.0309 | 0.0222 | 0.0077 | 0.0149 |
| *rps7* | 0.0204 | 0.0233 | 0.0124 | 0.0235 | 0.0173 | 0.0169 | 0.0146 | 0.0127 | 0.0131 | 0.0411 | 0.0126 | 0.0147 | 0.0203 | 0.0049 | 0.0226 | 0.0091 | 0.0010 | 0.0010 | 0.0117 | 0.0044 | 0.0107 | 0.0173 | 0.0271 | 0.0011 | 0.0205 | 0.0084 | 0.0285 |
| *rps8* | 0.0460 | 0.0380 | 0.0248 | 0.0248 | 0.0355 | 0.0357 | 0.0476 | 0.0396 | 0.0469 | 0.0334 | 0.0374 | 0.0365 | 0.0016 | 0.0236 | 0.0214 | 0.0256 | 0.0014 | 0.0040 | 0.0339 | 0.0222 | 0.0190 | 0.0193 | 0.0420 | 0.0619 | 0.0294 | 0.0221 | 0.0256 |
| *rps9* | 0.0195 | 0.0134 | 0.0228 | 0.0180 | 0.0094 | 0.0173 | 0.0257 | 0.0189 | 0.0230 | 0.0132 | 0.0277 | 0.0227 | 0.0014 | 0.0239 | 0.0163 | 0.0010 | 0.0249 | 0.0012 | 0.0518 | 0.0261 | 0.0011 | 0.0010 | 0.0160 | 0.0794 | 0.0130 | 0.0010 | 0.0164 |
| *tufA* | 0.0161 | 0.0249 | 0.0282 | 0.0210 | 0.0251 | 0.0211 | 0.0209 | 0.0147 | 0.0205 | 0.0245 | 0.0160 | 0.0199 | 0.0130 | 0.0145 | 0.0188 | 0.0137 | 0.0177 | 0.0115 | 0.0207 | 0.0170 | 0.0123 | 0.0121 | 0.0133 | 0.0130 | 0.0159 | 0.0151 | 0.0183 |
| *ycf4* | 0.0444 | 0.0137 | 0.0315 | 0.0428 | 0.0381 | 0.0182 | 0.0139 | 0.0219 | 0.0156 | 0.0257 | 0.0206 | 0.0191 | 0.0015 | 0.0250 | 0.0286 | 0.0135 | 0.0193 | 0.0211 | 0.0132 | 0.0011 | 0.0257 | 0.0303 | 0.0195 | 0.0024 | 0.0173 | 0.0013 | 0.0297 |
| Mean  value | 0.0151 | | | | | | | | | | | | 0.0161 | | | | | | | | | | | | | | |

Table S2 The LRT of branch model to identify the potentially fast-evolving genes.

| Gene | The dN/dS of colonial species | The dN/dS of unicellular species | lnL H0 | lnL HA | df | lnL 2*\|(HA-H0)\| | P value | FDR |
| --- | --- | --- | --- | --- | --- | --- | --- | --- |
| ***atpA*** | 0.0211 | 0.0378 | -14806.7991 | -14792.7256 | 1 | 28.1470 | 0.0000 | 0.0000 |
| ***rpl16*** | 0.0064 | 0.0318 | -3679.2970 | -3665.1593 | 1 | 28.2755 | 0.0000 | 0.0000 |
| ***psaB*** | 0.0132 | 0.0231 | -18914.3263 | -18903.5848 | 1 | 21.4830 | 0.0000 | 0.0001 |
| ***psbC*** | 0.0091 | 0.0217 | -10311.7703 | -10302.1337 | 1 | 19.2732 | 0.0000 | 0.0002 |
| ***atpB*** | 0.0173 | 0.0295 | -13479.9929 | -13472.0438 | 1 | 15.8981 | 0.0001 | 0.0007 |
| *ccsA* | 0.0472 | 0.0254 | -7481.0038 | -7473.6866 | 1 | 14.6343 | 0.0001 | 0.0012 |
| ***psbE*** | 0.0035 | 0.0269 | -1787.5071 | -1780.4864 | 1 | 14.0415 | 0.0002 | 0.0014 |
| ***psaJ*** | 0.0001 | 0.0235 | -1100.8388 | -1094.1318 | 1 | 13.4139 | 0.0002 | 0.0017 |
| ***psbA*** | 0.0118 | 0.0273 | -7072.3943 | -7066.6001 | 1 | 11.5882 | 0.0007 | 0.0041 |
| ***rps8*** | 0.0182 | 0.0389 | -4180.0185 | -4174.8878 | 1 | 10.2613 | 0.0014 | 0.0076 |
| *rps2* | 0.0626 | 0.0476 | -23127.1667 | -23122.5094 | 1 | 9.3147 | 0.0023 | 0.0116 |
| ***rpl2*** | 0.0189 | 0.0302 | -8568.5575 | -8564.0298 | 1 | 9.0552 | 0.0026 | 0.0122 |
| ***rps12*** | 0.0067 | 0.0166 | -3437.4909 | -3433.4879 | 1 | 8.0061 | 0.0047 | 0.0186 |
| ***rps14*** | 0.0170 | 0.0360 | -2976.0653 | -2972.0156 | 1 | 8.0994 | 0.0044 | 0.0186 |
| ***psbN*** | 0.0001 | 0.0085 | -856.1853 | -852.5782 | 1 | 7.2143 | 0.0072 | 0.0270 |
| ***atpI*** | 0.0183 | 0.0274 | -7402.7267 | -7399.5303 | 1 | 6.3927 | 0.0115 | 0.0401 |
| *psbM* | 0.0380 | 0.0134 | -826.9188 | -824.0742 | 1 | 5.6891 | 0.0171 | 0.0562 |
| *psbD* | 0.0104 | 0.0171 | -8276.9588 | -8274.1992 | 1 | 5.5191 | 0.0188 | 0.0585 |
| *rpl14* | 0.0064 | 0.0138 | -3360.1762 | -3357.5010 | 1 | 5.3504 | 0.0207 | 0.0611 |
| *rbcL* | 0.0174 | 0.0258 | -10293.4616 | -10290.9569 | 1 | 5.0095 | 0.0252 | 0.0706 |
| *psbF* | 0.0723 | 0.0225 | -650.4265 | -648.6117 | 1 | 3.6296 | 0.0568 | 0.1445 |
| *tufA* | 0.0359 | 0.0284 | -11025.1969 | -11023.3794 | 1 | 3.6349 | 0.0566 | 0.1445 |
| *psbH* | 0.0235 | 0.0434 | -2063.8227 | -2062.1239 | 1 | 3.3975 | 0.0653 | 0.1590 |
| *atpE* | 0.0166 | 0.0266 | -3324.5176 | -3322.8820 | 1 | 3.2712 | 0.0705 | 0.1595 |
| *atpF* | 0.0236 | 0.0344 | -5045.3442 | -5043.7166 | 1 | 3.2552 | 0.0712 | 0.1595 |
| *chlB* | 0.0242 | 0.0200 | -15283.3625 | -15281.8530 | 1 | 3.0190 | 0.0823 | 0.1707 |
| *chlN* | 0.0237 | 0.0191 | -12198.6675 | -12197.1497 | 1 | 3.0355 | 0.0815 | 0.1707 |
| *rps4* | 0.0200 | 0.0267 | -5955.5518 | -5954.2558 | 1 | 2.5921 | 0.1074 | 0.2148 |
| *psbK* | 0.0561 | 0.0276 | -1075.1896 | -1074.0051 | 1 | 2.3689 | 0.1238 | 0.2390 |
| *rpl20* | 0.0263 | 0.0361 | -3501.1748 | -3500.0809 | 1 | 2.1878 | 0.1391 | 0.2597 |
| *petB* | 0.0144 | 0.0105 | -5250.9544 | -5250.0007 | 1 | 1.9074 | 0.1672 | 0.3000 |
| *rps7* | 0.0185 | 0.0255 | -4259.0032 | -4258.0679 | 1 | 1.8704 | 0.1714 | 0.3000 |
| *psbZ* | 0.0523 | 0.0329 | -1686.6790 | -1685.9173 | 1 | 1.5234 | 0.2171 | 0.3377 |
| *rpl36* | 0.0272 | 0.0180 | -906.4768 | -905.6955 | 1 | 1.5627 | 0.2113 | 0.3377 |
| *rps18* | 0.0396 | 0.0523 | -3862.2332 | -3861.4556 | 1 | 1.5552 | 0.2124 | 0.3377 |
| *ycf4* | 0.0183 | 0.0242 | -4744.4606 | -4743.6569 | 1 | 1.6073 | 0.2049 | 0.3377 |
| *psbL* | 0.0001 | 0.0072 | -575.3634 | -574.6399 | 1 | 1.4470 | 0.2290 | 0.3466 |
| *rpl23* | 0.0474 | 0.0363 | -3085.1577 | -3084.4997 | 1 | 1.3159 | 0.2513 | 0.3704 |
| *psbJ* | 0.0386 | 0.0646 | -1099.4666 | -1098.8321 | 1 | 1.2689 | 0.2600 | 0.3733 |
| *cemA* | 0.0045 | 0.0067 | -5122.6788 | -5122.0679 | 1 | 1.2219 | 0.2690 | 0.3766 |
| *rps3* | 0.0485 | 0.0434 | -16206.3470 | -16205.7978 | 1 | 1.0984 | 0.2946 | 0.4024 |
| *petG* | 0.0080 | 0.0067 | -833.0265 | -833.5373 | 1 | 1.0216 | 0.3121 | 0.4162 |
| *rps19* | 0.0181 | 0.0234 | -2708.4649 | -2707.9812 | 1 | 0.9674 | 0.3253 | 0.4237 |
| *rps11* | 0.0177 | 0.0140 | -3516.3043 | -3515.8729 | 1 | 0.8628 | 0.3530 | 0.4492 |
| *petL* | 0.0086 | 0.0161 | -921.3762 | -921.0220 | 1 | 0.7084 | 0.4000 | 0.4869 |
| *psbI* | 0.0214 | 0.0101 | -679.3385 | -678.9732 | 1 | 0.7305 | 0.3927 | 0.4869 |
| *petD* | 0.0219 | 0.0258 | -4413.7961 | -4413.4776 | 1 | 0.6371 | 0.4248 | 0.5061 |
| *psaC* | 0.0094 | 0.0130 | -1717.2674 | -1717.0013 | 1 | 0.5323 | 0.4656 | 0.5432 |
| *petA* | 0.0337 | 0.0363 | -8531.7331 | -8531.5737 | 1 | 0.3187 | 0.5724 | 0.6542 |
| *psbT* | 0.0243 | 0.0332 | -648.3206 | -648.2113 | 1 | 0.2186 | 0.6401 | 0.7169 |
| *atpH* | 0.0036 | 0.0054 | -1443.8639 | -1443.7793 | 1 | 0.1692 | 0.6809 | 0.7476 |
| *clpP* | 0.0286 | 0.0299 | -12159.0816 | -12159.0146 | 1 | 0.1339 | 0.7145 | 0.7694 |
| *psbB* | 0.0212 | 0.0202 | -11609.7282 | -11609.6727 | 1 | 0.1109 | 0.7391 | 0.7809 |
| *chlL* | 0.0047 | 0.0049 | -6848.3712 | -6848.3603 | 1 | 0.0217 | 0.8829 | 0.8829 |
| *rpl5* | 0.0271 | 0.0263 | -5005.5354 | -5005.5225 | 1 | 0.0257 | 0.8726 | 0.8829 |
| *rps9* | 0.0335 | 0.0323 | -4043.7106 | -4043.6944 | 1 | 0.0323 | 0.8574 | 0.8829 |

Potentially fast-evolving genes are indicated in bold.

Table S3 The LRT of branch-site model to identify the potentially positively selected genes.

| Gene | lnL H0 | lnL HA | df | lnL 2*\|(HA-H0)\| | P value | FDR |
| --- | --- | --- | --- | --- | --- | --- |
| ***psaB*** | -18601.6212 | -18660.9855 | 1 | 118.7287 | 0.0000 | 0.0000 |
| ***psbB*** | -11476.8739 | -11507.3415 | 1 | 60.9351 | 0.0000 | 0.0000 |
| ***psbC*** | -10162.7833 | -10208.4296 | 1 | 91.2926 | 0.0000 | 0.0000 |
| ***rbcL*** | -10064.2712 | -10090.0867 | 1 | 51.6311 | 0.0000 | 0.0000 |
| ***tufA*** | -10688.4037 | -10734.0271 | 1 | 91.2468 | 0.0000 | 0.0000 |
| ***psbA*** | -7022.2349 | -7038.1916 | 1 | 31.9134 | 0.0000 | 0.0000 |
| ***rps4*** | -5944.5333 | -5955.5181 | 1 | 21.9695 | 0.0000 | 0.0000 |
| ***rpl5*** | -4987.5555 | -4995.8135 | 1 | 16.5161 | 0.0000 | 0.0003 |
| ***rpl16*** | -3671.6974 | -3679.2970 | 1 | 15.1992 | 0.0001 | 0.0006 |
| ***rps12*** | -3430.3255 | -3437.4909 | 1 | 14.3308 | 0.0002 | 0.0008 |
| ***atpF*** | -5034.7815 | -5040.9592 | 1 | 12.3554 | 0.0004 | 0.0022 |
| *atpE* | -3286.4163 | -3289.3140 | 1 | 5.7954 | 0.0161 | 0.0736 |
| *rps8* | -4173.8643 | -4176.0167 | 1 | 4.3048 | 0.0380 | 0.1608 |
| *psaC* | -1707.7400 | -1709.6493 | 1 | 3.8186 | 0.0507 | 0.1917 |
| *psbI* | -677.1459 | -679.0295 | 1 | 3.7671 | 0.0523 | 0.1917 |
| *atpH* | -1441.2534 | -1441.9947 | 1 | 1.4825 | 0.2234 | 0.7679 |
| *petD* | -4376.1266 | -4375.5959 | 1 | 1.0614 | 0.3029 | 0.9800 |
| *atpA* | -14373.4462 | -14373.4462 | 1 | 0.0000 | 1.0000 | 1.0000 |
| *atpB* | -13193.0169 | -13193.0169 | 1 | 0.0000 | 0.9989 | 1.0000 |
| *atpI* | -7343.5649 | -7343.5649 | 1 | 0.0000 | 1.0000 | 1.0000 |
| *ccsA* | -7346.9686 | -7346.9686 | 1 | 0.0000 | 1.0000 | 1.0000 |
| *cemA* | -5117.1487 | -5117.1486 | 1 | 0.0001 | 0.9930 | 1.0000 |
| *chlB* | -15119.1401 | -15119.1401 | 1 | 0.0000 | 1.0000 | 1.0000 |
| *chlL* | -6837.9812 | -6837.9813 | 1 | 0.0002 | 0.9888 | 1.0000 |
| *chlN* | -11883.9887 | -11883.9887 | 1 | 0.0000 | 1.0000 | 1.0000 |
| *clpP* | -11867.0807 | -11867.0807 | 1 | 0.0000 | 1.0000 | 1.0000 |
| *petA* | -8439.2855 | -8439.2855 | 1 | 0.0000 | 1.0000 | 1.0000 |
| *petB* | -5234.2173 | -5234.2234 | 1 | 0.0122 | 0.9119 | 1.0000 |
| *petG* | -833.0265 | -833.0265 | 1 | 0.0000 | 1.0000 | 1.0000 |
| *petL* | -921.3762 | -921.3762 | 1 | 0.0000 | 1.0000 | 1.0000 |
| *psaJ* | -1100.8388 | -1100.8388 | 1 | 0.0000 | 1.0000 | 1.0000 |
| *psbD* | -8180.5718 | -8180.5718 | 1 | 0.0000 | 1.0000 | 1.0000 |
| *psbE* | -1750.6927 | -1750.6927 | 1 | 0.0000 | 1.0000 | 1.0000 |
| *psbF* | -641.4416 | -641.4416 | 1 | 0.0000 | 1.0000 | 1.0000 |
| *psbH* | -1994.7325 | -1994.7325 | 1 | 0.0000 | 1.0000 | 1.0000 |
| *psbK* | -1075.1896 | -1075.1896 | 1 | 0.0000 | 1.0000 | 1.0000 |
| *psbL* | -575.3634 | -575.3634 | 1 | 0.0000 | 0.9989 | 1.0000 |
| *psbM* | -777.7583 | -777.7583 | 1 | 0.0000 | 1.0000 | 1.0000 |
| *psbN* | -856.1853 | -856.1853 | 1 | 0.0000 | 1.0000 | 1.0000 |
| *psbT* | -641.9448 | -641.9448 | 1 | 0.0000 | 1.0000 | 1.0000 |
| *psbZ* | -1686.6790 | -1686.6790 | 1 | 0.0000 | 1.0000 | 1.0000 |
| *rpl14* | -3358.8088 | -3358.8088 | 1 | 0.0000 | 1.0000 | 1.0000 |
| *rpl2* | -8535.9510 | -8535.9510 | 1 | 0.0000 | 1.0000 | 1.0000 |
| *rpl20* | -3472.5324 | -3472.5324 | 1 | 0.0000 | 1.0000 | 1.0000 |
| *rpl23* | -3085.1577 | -3085.1577 | 1 | 0.0000 | 1.0000 | 1.0000 |
| *rpl36* | -900.6097 | -900.6097 | 1 | 0.0000 | 1.0000 | 1.0000 |
| *rps11* | -3516.3043 | -3516.3043 | 1 | 0.0000 | 1.0000 | 1.0000 |
| *rps14* | -2926.2960 | -2926.2960 | 1 | 0.0000 | 1.0000 | 1.0000 |
| *rps18* | -3787.7903 | -3787.7903 | 1 | 0.0000 | 1.0000 | 1.0000 |
| *rps19* | -2695.9531 | -2695.9531 | 1 | 0.0000 | 0.9989 | 1.0000 |
| *rps2* | -22819.0189 | -22819.0189 | 1 | 0.0000 | 1.0000 | 1.0000 |
| *rps3* | -15982.2471 | -15982.2471 | 1 | 0.0000 | 1.0000 | 1.0000 |
| *rps7* | -4253.3156 | -4253.3156 | 1 | 0.0000 | 1.0000 | 1.0000 |
| *rps9* | -4017.3214 | -4017.3214 | 1 | 0.0000 | 1.0000 | 1.0000 |
| *ycf4* | -4731.9777 | -4731.9777 | 1 | 0.0000 | 0.9984 | 1.0000 |

Potentially positively selected genes are indicated in bold.
