# Supplementary material for: Evolutionary Analysis of Unicellular Species in Chlamydomonadales Through Chloroplast Genome Comparison With the Colonial Volvocine Algae
Source: Front Microbiol. 2019 Jun 18;10:1351. doi: 10.3389/fmicb.2019.01351 (PMC6591512; doi:10.3389/fmicb.2019.01351)
Supplement: Supplementary file 2 [file Table_2.DOCX]

## Materials and Methods of Photosynthetic Experiment

Two unicellular species were obtained from the FACHB collection. *Carteria* sp. FACHB-1856, *Chlamydomonas reinhardtii* FACHB-265 together with our six colonial species in this study were first grown in BG11 medium at 20-25°C under a 14 h light:10 h dark schedule under cool-white fluorescent lamps at an intensity of 1000-2000 lux. An aliquot of the culture was collected during the logarithmic growth phase, after centrifugated and removed the spent medium, the culture was diluted in a deficient BG11 (iron and calcium deficient). Then, the diluted cultures were inoculated to corresponding 100 mL medium to reach an optical density of 0.014 at 680 nm (OD_680_), measured in a UV-1780 spectrophotometer (SHIMADZU, Japan), there were four different treatments as follows: iron limitation BG11 under normal light (1000-2000 lux, BG11 without Ferric ammonium citrate), calcium limitation BG11 under normal light (1000-2000 lux, BG11 without calcium chloride), BG11 under high light (7000-8000 lux), BG11 under normal light (as control group). All treatment has three replicates. After cultured for five days, the Handy Plant Efficiency Analyzer apparatus (Handy PEA) with an attachment used for liquid samples (Hansatech Instruments Ltd., United Kindom) was used to measure the maximum quantum yields of PSII (Fv/Fm) at room temperature. The culture was dark-adapted completely for 10 min, the intensity of the excitation light was set to 1000 μmol m^-2^ s^-1^. Comparisons of the Fv/Fm were conducted using the Wilcoxon rank sum test as implemented in R.

## Results of Photosynthetic Efficiency

The Fv/Fm of unicellular and colonial species were list in Table S4, the original data were listed in Table S5. Under the calcium limitation BG11 treatment, we found only the unicellular species have grown up, all colonial algae died, which show that unicellular species are less sensitive to calcium limitation. By conduct the Wilcoxon rank sum test, we test whether Fv/Fm of iron limitation treatment less than control group, we found Fv/Fm of unicellular species show no statistical significance, and that of colonial species have statistical significance (P value < 0.05), which indicated that unicellular species are less sensitive to iron limitation. As our experimental procedure cannot remove the iron and calcium completely in the culture medium, there could be trace amount of iron and calcium left in culture medium. Here, we speculate that unicellular species could utilize trace amount of calcium and iron more efficiently, thus the limitation of iron or calcium have lower impact on unicellular species.

Compared the Fv/Fm between unicellular species and colonial species under different treatment, we found Fv/Fm of colonial species higher than unicellular species with statistical significance in control group (P value = 0.0178), and statistical significance under high light treatment (P value =0.0004). As the light get higher, the Fv/Fm difference of two group species gets bigger, we speculate unicellular species could utilize less light than colonial species, one possible reason could be the unicellular species may have lower demand for light.

Table S4 Fv/Fm of two unicellular species and six colonial species under different treatments. Fv/Fm value are expressed as mean ± SD. Bold indicates P value less than 0.05.

|  | Unicellular species | Colonial species | P value  (unicell less than colonial) |
| --- | --- | --- | --- |
| Control group | 0.78±0.04 | 0.82±0.05 | **0.0178** |
| Iron limitation | 0.76±0.04 | 0.78±0.05 | 0.2022 |
| High light | 0.78±0.02 | 0.85±0.05 | **0.0004** |
| P value  Iron limitation less than control group | 0.1201 | **0.0141** |  |
| P value  High light higher than control group | 0.5314 | 0.0692 |  |

Table S5 The Fv/Fm of two unicellular species and six colonial species under different treatment.

|  | Replicates | *Chlamydomonas reinhardtii* | *Carteria* sp. | *Colemanosphaera charkowiensis* | *Volvulina compacta* | *Pandorina colemaniae* | *Pandorina morum* | *Colemanosphaera angeleri* | *Yamagishiella unicocca* |
| --- | --- | --- | --- | --- | --- | --- | --- | --- | --- |
| Control group | 1 | 0.811 | 0.727 | 0.843 | 0.741 | 0.8489 | 0.8867 | 0.78 | 0.872 |
|  | 2 | 0.817 | 0.751 | 0.841 | 0.765 | 0.8403 | 0.855 | 0.763 | 0.874 |
|  | 3 | 0.818 | 0.75 | 0.84 | 0.751 | 0.839 | 0.8923 | 0.78 |  |
| Iron limitation | 1 | 0.785 | 0.696 | 0.82 | 0.688 | 0.77 | 0.862 | 0.778 | 0.762 |
|  | 2 | 0.798 | 0.742 | 0.827 | 0.701 | 0.755 | 0.862 | 0.765 | 0.749 |
|  | 3 | 0.792 | 0.726 | 0.836 | 0.754 | 0.763 | 0.884 | 0.77 | 0.758 |
| High light | 1 | 0.785 | 0.767 | 0.81 | 0.813 | 0.845 | 0.8833 | 0.883 | 0.936 |
|  | 2 | 0.799 | 0.772 | 0.803 | 0.817 | 0.847 | 0.886 | 0.925 | 0.843 |
|  | 3 | 0.809 | 0.769 | 0.8 | 0.804 | 0.843 | 0.9283 |  | 0.865 |
